# Artificial intelligence to enhance the diagnosis of ocular surface squamous neoplasia

**DOI:** 10.1038/s41598-025-94876-4

**Published:** 2025-03-20

**Authors:** Kincső Kozma, Zoltán Richárd Jánki, Vilmos Bilicki, Adrienne Csutak, Eszter Szalai

**Affiliations:** 1 Department of Ophthalmology, Medical School, University of Pécs, Rákóczi u. 2, Pecs, 7623 Hungary; 2 Department of Software Engineering, University of Szeged, Dugonics tér 13, Szeged, 6720 Hungary

**Keywords:** Artificial intelligence, In vivo confocal microscopy, Ocular oncology, Ocular surface squamous neoplasia, Neural network, Cancer imaging, Computational science

## Abstract

To provide an artificial intelligence (AI) method using in vivo confocal microscopy (IVCM) to differentiate ocular surface squamous neoplasia (OSSN) from other lesions and compare the performance of well-known AI-related solutions. A dataset of 2,774 IVCM images, comprising OSSN and other ocular surface diseases was used to train three deep learning models: ResNet50V2, Yolov8x, and VGG19. These models were trained to identify OSSN-related lesions by recognizing specific visual features, including the “starry-sky” pattern, hyperkeratosis, mitotic figures and irregularly shaped epithelial cells. To mitigate class imbalance, a novel square-based data augmentation strategy was employed. Additionally, we implemented a few-shot learning model to enhance the precision of rare symptoms, such as mitosis. To enhance model interpretation, Shapley values and Uniform Manifold Approximation and Projection (UMAP) analysis were employed to explain decision-making processes. The AI models demonstrated high accuracy in distinguishing healthy tissues from pathological ones, achieving over 90% accuracy across all models. In our binary classification task, all AI models had accuracy above 97% (precision ≥ 98%, recall ≥ 85%, F1 score ≥ 92%). The model achieved lower accuracy in 4 class labeled classification. Aggregation of cell-level results provided the best performance with an F1 score of 100%. The models successfully identified patient-specific features in IVCM images, suggesting that these images can act as “fingerprints”. Our AI model utilizing IVCM was able to classify OSSN with high accuracy. Moreover, cell-level classification results could be backpropagated to image-level and patient-level. The patient-specific information within IVCM images offers promise for personalized diagnostics and treatment monitoring in ocular oncology.

## Introduction

Ocular surface squamous neoplasia (OSSN) compromises a spectrum of benign, premalignant and malignant epithelial tumors of the conjunctiva and cornea^1^. Excision biopsy with no touch technique and cryotherapy followed by histopathology is the gold standard for diagnosis and subtyping of OSSN for appropriate management^2^. However, non-invasive imaging modalities such as anterior segment optical coherence tomography (AS-OCT) and in vivo confocal microscopy (IVCM) are of diagnostic importance by providing high-resolution optical biopsies of the ocular surface^3^.

IVCM is an imaging technique that can evaluate the conjunctiva and cornea at a cellular level and may provide a presumptive or definitive diagnosis of different ocular surface diseases. IVCM can also provide real-time imaging of the tumor and surrounding tissue allowing for accurate and immediate diagnosis. This can lead to earlier detection and treatment, which is associated with better outcomes for patients. The technology allows for a real-time, in vivo and high-resolution optical biopsy of ocular surface lesions with a high sensitivity and specificity^4^.

Recent advancements in artificial intelligence (AI), specifically deep learning (DL) through the use of convolutional neural networks (CNNs) have shown significant promise in revolutionizing medical image analysis and disease diagnosis. This is particularly relevant in ophthalmology where CNNs have demonstrated remarkable success in image classification tasks leading to more accurate diagnoses, personalized treatment plans, and improved monitoring of treatment outcomes in ocular oncology^5,6^. One of the most significant challenges is the need for large datasets of images to train AI algorithms. AI can assist in the differentiation between benign and malignant lesions of the ocular surface. OSSN can look similar to other non-cancerous conditions on the ocular surface which can make diagnosis challenging^7^. AI algorithms can be trained to recognize the specific characteristics of OSSN and differentiate it from other conditions. The primary goal of researchers is to develop an AI-based system capable of identifying and classifying multiple types of ocular surface lesions at an early stage. Another aim is to early diagnose malignancies of the eye by using AI algorithms that may reduce the need for surgeries and biopsies^8^.

The purpose of this study was to develop and test AI algorithms that can differentiate OSSN from other ocular surface diseases based on specific characteristics. The secondary focus of this study is on model interpretability, a critical factor for clinical adoption of AI systems. By employing Shapley values and UMAP, we aim to provide transparency into the decision-making processes of our models. In order to confirm the results and implications of our Shapley analysis, we conducted Grad-CAM and feature importance analyses as well.

## Methods

### Imaging, preprocessing and labeling methodology

IVCM images were acuired using Heidelberg Retina Tomograph II Rostock Cornea Module (HRT II RCM; Heidelberg Engineering GmbH, Heidelberg, Germany). The snapshots are gray-scaled and saved in the Joint Photographic Experts Group (JPEG) format. To ensure dataset integrity, images with flaws such as blurriness, poor contrast, or artifacts obscuring diagnostic features were excluded. Specific criteria included clear visibility of cellular structures, absence of motion blur, and adequate illumination. The images have a resolution of 384 × 484 pixels, along the bottommost 384 × 100 area includes meta-information about the image. The section of meta-information has been removed to distinguish between signs and pathologies based on the cells, cell groups, and their pictorial features, no other modifications were made to the images. Therefore, we abandoned the possibility that the neural networks acquire the information contained in the footer; consequently, the cells are the sole focus. The final resolution of each IVCM image was 384 × 384 pixels after the footer was removed.

**Fig. 1:**
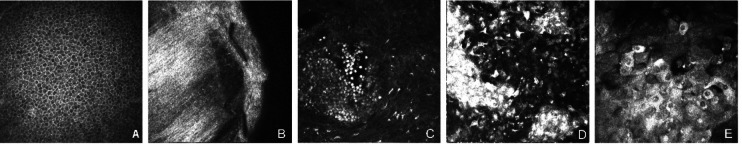
**A.** Normal epithelial cell pattern on IVCM image. **B.** Pterygium **C.** Keratitis **D**. Melanoma **E**. OSSN.

The final dataset comprised 2,774 IVCM images, categorized into five classes: OSSN (745 images), normal cornea (1,559 images), melanoma (270 images), pterygium (115 images), and keratitis (85 images). Each patient contributed an average of 27.7 images. Patient-specific allocation ensured no overlap between training and validation datasets to prevent overfitting and model reliance on patient-specific features. In the OSSN group were included 745 images containing OSSN lesions, the non-OSSN group comprised conditions like pterygium, keratitis, conjunctival melanoma and normal cornea, non-OSSN lesions were present in 2,029 images (Fig. 1).

**Fig. 2:**
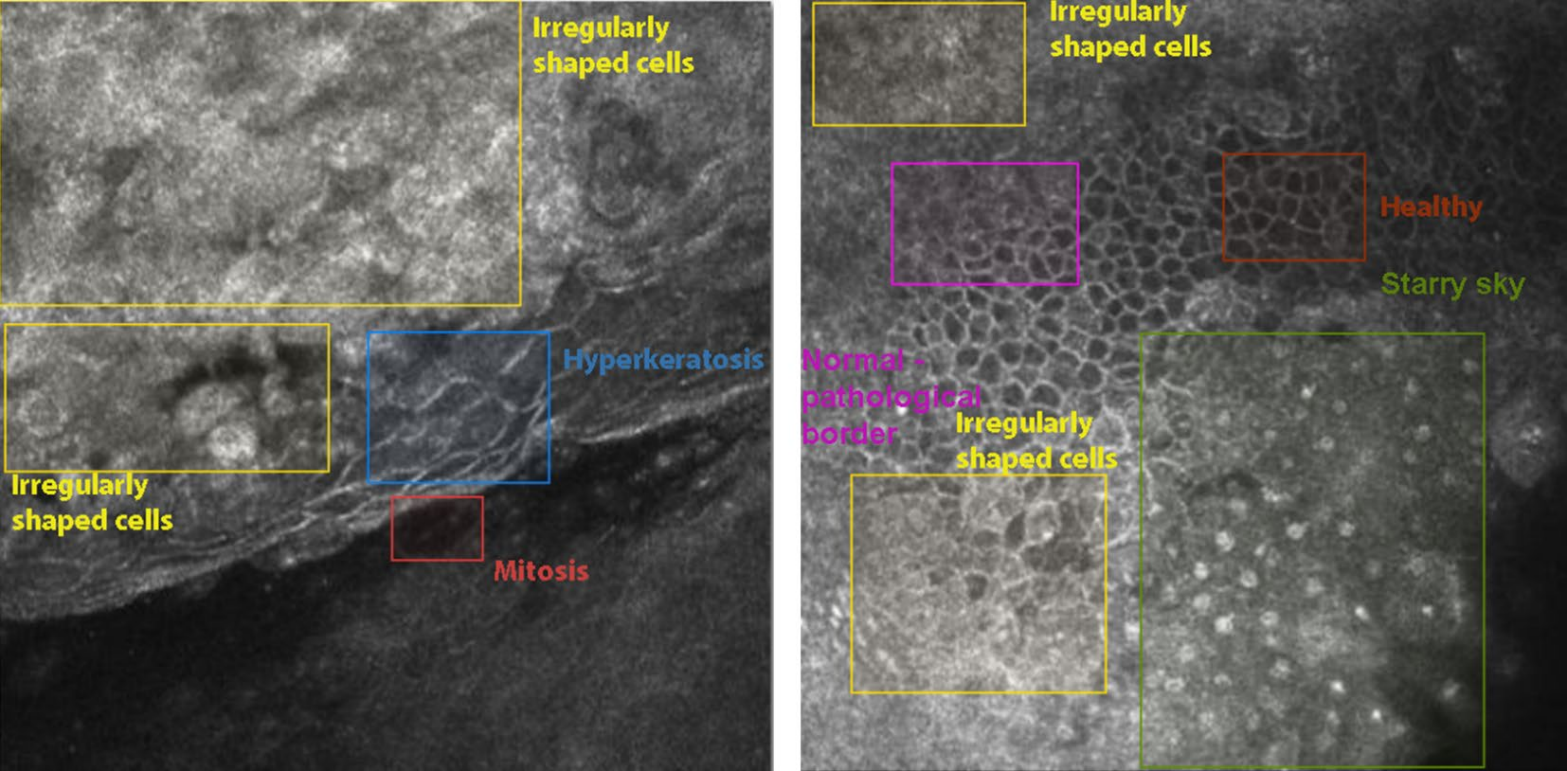
The most common OSSN-related clinical signs.

Then images can be classified by utilizing the entire image or identifying the most influential components. It is reasonable to identify OSSN or its suspicion by identifying its visual features, as the suspicion of OSSN is primarily based on the presence of visible pathological signs. It might seem to be a problem with feature detection and classification. Consequently, we compiled a list of OSSN-related pathological alterations („starry-sky” pattern, hyperkeratosis, mitotic figures and irregularly shaped epithelial cells) and labeled the images and the sign-related areas. The malignant regions were manually identified using the following criteria: 1) starry-sky pattern (hyperreflective nucleus), 2) hyperkeratosis, 3) mitotic figures, and 4) enlarged, irregular epithelial cells, Fig. 2. shows sample images selected from the different signs. The images and their subregions were labeled using the Computer Vision Annotation Tool (CVAT)^9–11^.

It is challenging to ensure that there is no overlap between two images when creating IVCM images. As a result, we divided the training and validation sets according to the patients involved to prevent the results from being distorted by previously observed cell groups.

The images were not subjected to any additional image manipulation procedures.

All IVCM images used for creating the training and test datasets were collected at the University of Pécs, Department of Ophthalmology under institutional review approved by University of Pécs Ethics Committee (No. 8127 - PTE 2019, KK/93 − 1/2020) and after informed consent from patients.

## Classification and analysis of results

An aim of this study was to compare and analyze well-known AI-related solutions to ascertain the rationale behind their decisions and create a functional AI solution for OSSN identification. To conduct a thorough investigation, we have chosen three distinct neural networks for transfer learning: Yolov8x^12^, ResNet50V2^13^, and VGG19^14^. Three deep learning models were selected for their proven efficacy in medical image analysis: ResNet50V2: A residual network optimized for feature learning and gradient flow. Yolov8x: Designed for object detection and feature localization. VGG19: A deep CNN known for its robustness in image classification tasks. In order to achieve analogous outcomes, we trained the neural networks on the same dataset and employed identical parameters and configurations for the image sizes, training properties, and augmentations. In the context of augmentation, we implemented a 10-degree rotation, as well as scaling and flipping with a 50% probability. The pace of prediction was not an important aspect of this study, as our objective was to achieve the highest possible accuracy in OSSN detection, rather than the quickest solution. The macro-average values refer to the unweighted average of performance metrics across classes, including accuracy, precision, recall, and F1-Score, which serve as the basis for comparisons.

## Deep learning models for classification tasks

After manual evaluation of IVCM images, we found that patients with healthy cells can be certainly differentiated from the pathological lesions. However, different lesions may exhibit similar pictorial features. This makes OSSN identification challenging even for experts and it often requires further examinations. To prove our statement, we trained different neural networks with a variety of purposes. Here, we list the tasks completed that produced new results in the field of ophthalmology and artificial intelligence as well.

- Differentiating healthy and unhealthy IVCM images.
- Classification of OSSN-related signs.
- Showing that IVCM images may contain patient-specific features.
- Classification of cells with high accuracy.

## Differentiating healthy and OSSN-diagnosed IVCM images

As a first step in the classification problem, we validated whether AI can distinguish healthy cells from pathological changes. Healthy and abnormal patterns are easily distinguishable. However, we may have to modify our AI methodologies and approach because our final goal is not trivial visually. Also, this task validated that we can perform a good classification with a small number of samples if the patterns are visually different.

In this classification task, we used 1,559 healthy and 277 OSSN-diagnosed images. In order to prevent the acquisition of patient-specific features, the training and validation data have been split such that a patient who is included in the training set is not included in the validation set. Hence, we can prevent not only the phenomenon of overfitting, but the learning of patient- and image-specific features as well.

We selected multiple models to make a good comparison and find the best performing one. We compared the ResNet50V2, Yolov8x and VGG19 models for achieving our classification problem. All three models are complex enough to operate on complex problems with high accuracy. ResNet50V2 is comprised of 50 convolutional layers and 23 million hyper-parameters that are meticulously organized. The largest variant in the Yolov8 family is the Yolov8x. It contains over 130 million hyper-parameters. VGG19 comprises 19 layers and more than 140 million trainable parameters. With these capabilities, they are capable of learning the most intricate details and abstract features, and they are suitable for a variety of inputs.

## Classification of OSSN-related signs

The primary challenge is to differentiate the pathological signs from other non-OSSN-related features. Often the different pathologies-related signs have similar visual features. However, OSSN has a well-defined list of pathological features. Since the alterations have visible features on IVCM images, a potential solution is to detect and classify these features on IVCM images. We have annotated the 4 most common OSSN-related clinical signs which are hyperkeratosis, starry sky, mitosis, irregularly shaped cells and trained the neural networks using the annotated images. Rectangle-shaped labeling was our favored method since the annotations were prepared for classification. We trained various classifiers to validate if a feature-based approach can be efficient in an OSSN identification problem.

It is known that the more samples are available in a classification problem, the better and more generic solution can be obtained. In a multi-class classification problem, it is even more crucial to have several samples in each class. We realized that our dataset is quite unbalanced. Some features are only present in a few images. It is important to note that mitosis is a quite rare sign, and this rareness was present in our dataset as well. Furthermore, we have a quite small database including images from less than 100 patients only.

**Fig. 3:**
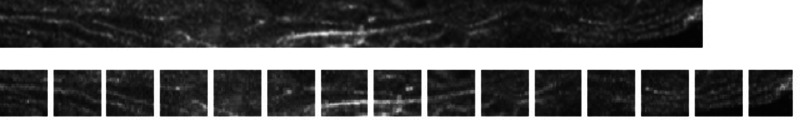
Increasing the dataset by splitting a rectangle showing hyperkeratosis into squares.

Because of the small dataset available, we introduced a new approach to increase the number of samples. Most of the OSSN-related signs are defined by regions of the ocular surface. Nonetheless, these regions can be subdivided into smaller subregions where the distinguishing features remain identifiable. The affected areas consist of homogeneous cell types, which ensures that aggregating cell-level results to the image level does not introduce distortion into the overall findings. Naturally, the confidence is higher if bigger regions can be seen in one piece even by experts. If we split the rectangles into squares that describe the alteration, we can still find the features on the images but the number of samples can be increased with minimal overlap (Fig. 3).

We divided the rectangles into squares in a way that the dimensions were determined by the shortest side of the original size of the rectangle. Thus, each sign - if its annotation is not described by a square - can be sampled with at least 2 squares. So, each annotation can be divided into

1 $$\varvec{\vert} \rm max\left( {wi},{hi} \right)min\left( {wi},{hi} \right)\varvec{\vert}$$

parts. Based on Eq. (1), we have to take into account that the squares created from the same annotation must be in the same training or validation dataset because the last generated square image may overlap with the previous one.

**Fig. 4:**
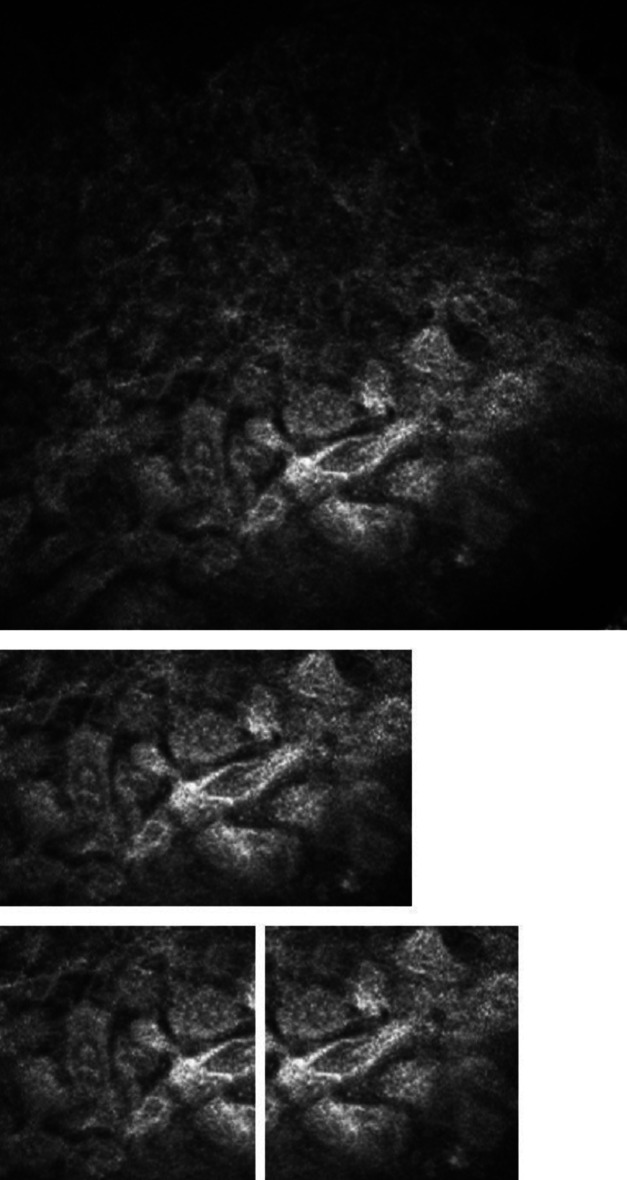
Splitting an annotation into squares.

During the annotation phase (Fig. 4), we successfully labeled 538 regions with rectangles and after the square-based splitting, we had 1,236 images, so the square-based splitting produced a more than 2 times larger dataset for training and validation. However, it is important to note that remarkable class imbalances can be observed, mainly for the symptom of mitosis. Our symptom classifier was weaker than the other classifiers, so we trained models for a binary classification problem to see whether the labeled squares could be identified as OSSN-related alterations or not, so we divided the dataset into two classes, OSSN and other. Nevertheless, to address this issue of dataset imbalance, we implemented and validated a few-shot learning approach that performs well in case of rare symptoms as well.

### Few-shot learning

It is crucial to evaluate the prevalence of the various OSSN-related symptoms when discussing the clinical relevance of our findings. There are symptoms that are considered to be typical of OSSN; however, they are rare and difficult to obtain samples of. Furthermore, the application of distinct techniques to each task is logical due to the intricacy of OSSN-related sign classification. Mitosis is a feature of this nature; however, none of the models were able to accurately classify the validation data of it due to the limited number of samples obtained. To address this challenge, we explored the use of few-shot learning, utilizing easyfsl^15^ to enhance classification performance. ResNet50 served as the base for our model, and we applied a leave-one-out cross-validation approach. Additionally, our square-based augmentation technique was implemented to optimize the training data and improve model performance.

## Finding patient-specific features on IVCM images

In a recent research^16^, it was shown that the human body carries features that might be person-specific and these visual appearances may distort the results of an AI if the machine focuses on them. Although we are analyzing the images at a low level, it is still possible that person-specific features can be identified in these images. Results gained in this field not only present novelty in IVCM image analysis but highly influence the data split strategy as well. As we mentioned before, during the imaging process, images may overlap. In this task, we carefully selected the training and validation datasets to guarantee there is no overlap between the two images. We selected the images including both healthy ones and pathological alterations. The selected patients had the most non-overlapping images in our dataset. We successfully split our dataset based on the involved patients and made classifications for both healthy and unhealthy images in which the class labels were the patients themselves.

We selected 142 images from 9 patients including only pathological alterations and then validated the approach on healthy images as well involving 11 patients and 62 images. These patients had the most non-overlapping images in both tasks. In these classification tasks, we expected to see the correlations between the patients and their images. So if the average metrics of the neural networks are above 50%, we can say that the images hold patient-specific information that influences the decision-making of the neural networks. Since it has already been shown at a higher level^17^, we assumed that this effect might be present at a lower level as well, even at the level of cell groups. Furthermore, we can show how these features affect the results of the different types of neural networks.

We have tested the hypothesis with three different neural networks. We trained them with the same dataset with 100 epochs. Regarding the training configurations and augmentations, there were no differences among the models. All models were trained and evaluated with a categorical approach.

## Classification of cells

IVCM images provide information about cells in different layers of the ocular surface structures. Experts rarely examine cells separately because they seem to be less informative than analyzing them in groups. However, AI may find cell-specific features that allow cells to provide information on their own. At the level of cells, we still have a group of pixels that may provide information about the image and the patient. Also, the level of cells is the lowest possible level of analysis that can be interpreted by an expert as well.

Cell-level annotation is a time-consuming and often difficult task for human experts as well, so we automatized the labeling with segmentation and made only expert-based validation. We used the Segment Anything^18^ model (SAM) created by Meta to segment the different types of cells found on an image. We did not train SAM for finding the cells, the automatic segmentation tool found the well-identifiable cell images that did not require further manual corrections. With SAM, we classified the cells into two groups, the OSSN-related and non-OSSN-related cells. Here, it is important to note that the group of non-OSSN-related cells contained various cells from the regions of elastic fibers, leukocytes and melanocytes. So, neither the training nor the validation set contained cells from healthy regions. All segmented cells were validated by experts based on cell morphology and pathological features, and finally,

we kept 7,501 images. 5,111 cell images were classified as OSSN-related cells and 2,390 images were considered as non-OSSN-related cells. Table 1. summarises the number of images used across different analysis stages.

**Table 1:** Number of images used across different analysis stages.

Stage/Task	Image Description	Number of Images	Purpose
Initial Dataset	High-quality images selected, after quality screening	2,774	Comprehensive collection of OSSN and non-OSSN images, Removed flawed/blurred images; resized to 384 × 384 pixels.
Training Binary Classification Models	Healthy vs. pathological classification	1,836 (1,559 healthy + 277 OSSN)	To test models for binary classification of health status.
OSSN Sign Classification	Annotated OSSN-related signs	1,236 (rectangles divided into squares)	Identify specific pathological signs in images.
Cell-Level Classification	Segmented cells (OSSN vs. non-OSSN)	7,501 (5,111 OSSN + 2,390 non-OSSN)	Detailed classification at cellular level for improved accuracy.

After having a larger and validated dataset, we trained the 3 selected neural networks for the binary classification problem with 100 epochs.

### Aggregation of cell-level

We made further aggregations and showed that this approach can be successful even with a small dataset. In our work, we defined three levels: these are the cell level, the image level, and the patient level, respectively. The relationship between the three levels can be described by the logical formulas of Eqs. 2–3. Collecting the classification results of the segmented cells in a given image, we can say that if at least 50% of the cells belong to the OSSN category, then we label the image with OSSN. Elevated to the third level, if at least half of the patient’s images are labeled OSSN, then we label the patient with OSSN as well.

2 $$\:OSSN\left(k\right)\:\iff\:\:(\frac{{o}_{{k}_{b}}}{{n}_{{k}_{b}}}\ge\:0.5)$$

where k is the given image of patient b

3 $$\:OSSN\left(b\right)\:\iff\:\:(\frac{{p}_{b}}{{m}_{b}}\ge\:0.5\:\wedge\:\:{p}_{b}=|{k}_{b}\left|OSSN\right({k}_{b}\left)\right|)$$

where k is the given image of patient b

### Evaluation on external IVCM datasets

To assess the generalizability of our model, we identified external IVCM datasets named CORN-1, CORN-2, CORN-3, and CORN1500, which were recently published^19^. These datasets contain a total of 5,038 images depicting corneal nerve fibers. Although these datasets were originally designed for different research purposes, they are suitable for validating our classifiers.

Since the images in these datasets were captured at a lower magnification, cell-level classification was not feasible, as individual cells were not visible. However, we were able to evaluate our symptom-level classifier on these datasets to determine its effectiveness in a broader context.

### Shapley values

Shapley values^20^, a concept borrowed from cooperative game theory, have emerged as a pivotal tool in the realm of explainable artificial intelligence (XAI), particularly within the domains of neural networks and image classification. These values provide a rigorous, equitable method for attributing the contribution of each input feature towards the output of a model, allowing for a deeper understanding of how complex models, such as deep neural networks, make decisions. In the context of image classification, Shapley values dissect the model’s decision-making process by quantifying the impact of each pixel or segment of the image on the final classification result. This method stands out for its ability to offer a mathematically grounded and fair explanation, adhering to properties like efficiency (the total contribution of all features equals the output of the model) and symmetry (equal contribution leads to equal attribution). SHapley Additive exPlanations (SHAP)^21,22^ is an implementation of the game theoretic approach to explain the output of machine learning models. We have evaluated our best trained AI model and generated all cell images with this tool.

### Dimension reduction

To validate the results and understand the complexity of the problem, we introduced the so-called Uniform Manifold Approximation and Projection (UMAP) technique^23^. It is an advanced, non-linear dimensionality reduction technique that plays a crucial role in the field of explainable AI, particularly in the context of neural networks and image classification tasks. UMAP facilitates the understanding and interpretation of complex, high-dimensional data by projecting it onto a lower-dimensional space, preserving the essential topological and structural relationships of the original data. This property is invaluable for neural networks that process image data, where the high dimensionality of inputs (i.e., images) often obscures the underlying patterns and features relevant to classification tasks.

In UMAP projection, one of the key parameters is the so-called metric that controls how distance is computed in the ambient space of the input data. UMAP offers a wide list of metrics, like Minkowsky-style, miscellaneous spatial, normalized spatial, angular and correlation metrics and metrics for binary data. We made analyses with a number of metrics, like the Hamming and Yaccard distance functions, the Yule distance function that compares two binary vectors (e.g. u and v) using Eq. 4. and the Russell-Rao distance function which compares two binary vectors (e.g. u and v) using Eq. (5)^24–27^.

4 $$\:{D}_{Y}=\frac{2*{c}_{TF}*{c}_{FT}}{{c}_{TT}*{c}_{FF}+{c}_{TF}*{c}_{FT}}$$

where c_ij_ is the number of occurrences of u[k] = i and v[k] = j for k < n.

5 $$\:{D}_{RR}=1-\frac{{c}_{TT}}{n}$$

where c_ij_ is the number of occurrences of u[k] = i and v[k] = j for k < n.

### Grad-CAM and feature importance analysis

The Shapley values from our previous analysis were relatively low, which may raise concerns about their validity. In order to verify the accuracy of our analysis and its implications, we implemented two additional methodologies to assess our neural network that had been trained. A direct, gradient-based approach to visualization is employed by the Gradient-weighted Class Activation Map (Grad-CAM)^28^ to identify regions of immediate significance in the model’s decision-making process. It distinguishes between relevant and non-relevant regions by emphasizing regions with high activation in the final convolutional layers in a manner that is binary in nature. Immediacy and interpretability are the hallmarks of this approach.

In order to conduct a thorough examination of our neural network, we additionally performed a feature importance analysis^29^. It employs a more systematic method, allocating individual significance scores to each feature on an individual basis. Through techniques such as ablation studies or permutation importance, this method quantifies the contribution of each feature to the model’s overall performance.

## Results

### Classification results of healthy and unhealthy IVCM images

In our first binary classification task all three models (Resnet50V2, Yolov8x and VGG19) had an accuracy of above 90%. (Table 2) Both ResNet50V2 and Yolov8x showed 99% of F1-Score, while VGG19 produced more false negative values that reduced the F1-Score value significantly.

**Table 2:** Classification macro-average value results of healthy and unhealthy IVCM images.

Model / Metrics	Accuracy	Precision	Recall	F1-Score
ResNet50V2	98%	98%	100%	99%
Yolov8x	99%	99%	99%	99%
VGG19	97%	99%	85%	92%

Based on the results, we further improved the base models for the more complex classification problems.

### Classification results of OSSN-related signs

By splitting the rectangle annotations into squares we improved our training and validation results a lot but the average precision of the model was still around 50% (Table 3). During evaluation of the results, we found that neural networks confuse the classes of irregularly shaped cells and the “starry sky” pattern. Furthermore, none of the models hit the correct class for the samples of mitosis. Based on the results, we found that our dataset is too small and unbalanced to train a general neural network for this 4-class classification problem even using special augmentation techniques and applying proper weights. We realized that we must simplify the problem or we should go to a lower level to obtain acceptable results.

If we consider the OSSN-related signs task as a binary classification problem, and merge the different symptoms, we can achieve significantly better results. We collected all OSSN-related symptoms into one group and the non-OSSN-related symptoms formed the other group. With this approach, we have a ResNet50V2 model with an F1-Score of 92%, the Yolov8x has an F1-Score of 95%. VGG19 has the lowest F1-Score in this task, but it is still acceptable with the value of 89%.

Although, in the separate classification of OSSN-related symptoms, our models did not perform well, it is important to note that we propose solutions for addressing the issues related to the imbalanced dataset in Section .

**Table 3:** Classification results of OSSN-related signs.

Models / Metrics	Accuracy	Precision	Recall	F1-Score
ResNet50V2	95%	97%	88%	92%
Yolov8x	97%	98%	93%	95%
VGG19	93%	91%	87%	89%

### Few-shot learning

Our concept was validated using easyfsl, which effectively classified the mitotic samples but made false predictions for the other classes. Consequently, we made adjustments to a few-shot learning model. The leave-one-out cross-validation results showed that the model reliably classified mitosis at 100% and correctly classified 78% of the other symptoms. These findings indicate that integrating the binary symptom-level classifier with the few-shot learning model can substantially enhance the generalizability of our OSSN diagnosis approach.

### Classification results of patient-specific images

All three models showed a good performance in the classification of patient-specific OSSN-diagnosed images (Table 4). The highest F1-Score value was produced by ResNet50V2, so that is the model that might focus on patient-specific features the most from the 3 trained models. Meanwhile, VGG performed 14% worse in this task.

**Table 4:** Classification results of patient-specific OSSN-diagnosed images.

Models / Metrics	Accuracy	Precision	Recall	F1-Score
ResNet50V2	92%	64%	67%	65%
Yolov8x	89%	60%	57%	56%
VGG19	86%	55%	51%	51%

we have seen that OSSN-diagnosed images May hold patient-specific features but this phenomenon might be true for the healthy images. During the selection of the healthy images, it was more difficult to find non-overlapping images for training and validation. we have fewer images here but the results still show a similar phenomenon. ResNet50V2 gave the best and VGG19 showed the worst results in the healthy patient classification task. However, the differences are much higher among the models here. Based on these results, ResNet50V2 seems to be the most sensitive to patient-specific features (Table 5). Yolov8x and VGG-19 can be less sensitive, they May perform better on images that are more difficult to assign to specific patients.

**Table 5:** Classification results of patient-specific healthy images.

Models / Metrics	Accuracy	Precision	Recall	F1-Score
ResNet50V2	95%	74%	73%	70%
Yolov8x	93%	55%	54%	51%
VGG19	90%	45%	54%	45%

### Classification results of cells

**Fig. 5:**
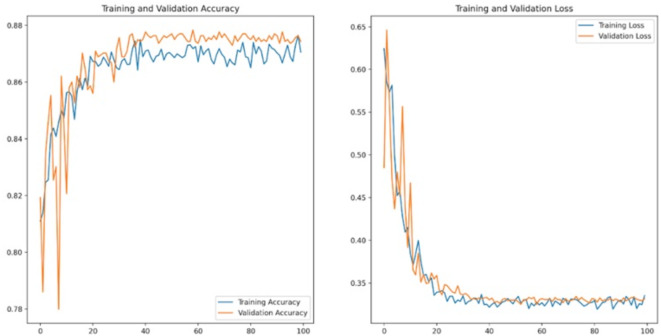
The accuracy and loss curves of VGG19.

All 3 models produced higher than 85% of F1-Scores. The best results were obtained by the VGG19 model with 90% of F1-Score. As the accuracy and loss curves of VGG19 are shown in Fig. 5., the model did not overfit, so the training process was successful. Table 6. concludes the results of the models. Based on the results, the models trained for cell-level classification can produce the most stable results in the OSSN identification problem.

**Table 6:** Classification results of OSSN-labeled cells.

	Accuracy	Precision	Recall	F1-Score
ResNet50V2	88%	87%	84%	85%
Yolov8x	90%	92%	86%	88%
VGG19	92%	92%	88%	90%

### Aggregation of cell-level results

The accuracy of our models was further enhanced by the aggregation of the results derived at the cell-level, as illustrated in Table 7. The F1-score of each of the three models exceeds 90%. Therefore, it is possible to differentiate the OSSN images from other IVCM images that were not classified as healthy as a result of the precise cell-level classification. In this instance, the metrics of the models are exceedingly similar.

**Table 7:** Results of image-level classification.

First level of aggregation (cells)	Accuracy	Precision	Recall	F1-Score
ResNet50V2	95%	97%	90%	93%
Yolov8x	95%	97%	89%	92%
VGG19	95%	96%	91%	93%

The models were reassessed following the second aggregation (see Table 8). the patient is classified at this level based on the aggregated image-level results. We can observe that ResNet50V2 and VGG19 attained a 100% F1-Score on this assignment. However, Yolov8x experienced a slight decrease. In summary, Yolov8x committed more substantial errors during the cell-level classification process, which had significant impacts on the patient level.

**Table 8:** Results of patient-level classification.

Second level of aggregation (images)	Accuracy	Precision	Recall	F1-Score
ResNet50V2	100%	100%	100%	100%
Yolov8x	92%	95%	88%	90%
VGG19	100%	100%	100%	100%

#### Evaluation on external IVCM datasets

The evaluation of our symptom-level classifier on the external IVCM datasets yielded an F1-Score of 86.4%, demonstrating strong performance despite differences in image characteristics. This result highlights the potential clinical applicability and robustness of our approach across diverse datasets, supporting its generalizability in real-world settings.

### Shapley values

Our visual experiences showed that healthy cells can be easily distinguished from unhealthy ones. However, after analyzing the cropped cells, we found that it is not an easy task to determine if an unhealthy cell is an OSSN-related cell or not. Since cells are very simple imaging creatures, they consist of few pixels, and they do not have many features, we cannot find many decision points in their classification problem. Most of the machine learning models provide only results, but there are no justifications behind them. Here, we found that our trained neural network can efficiently distinguish OSSN-related cells from other sickness-related cells, but it is not obvious which pixels or pixel groups played the most important role in classification.

**Fig. 6:**
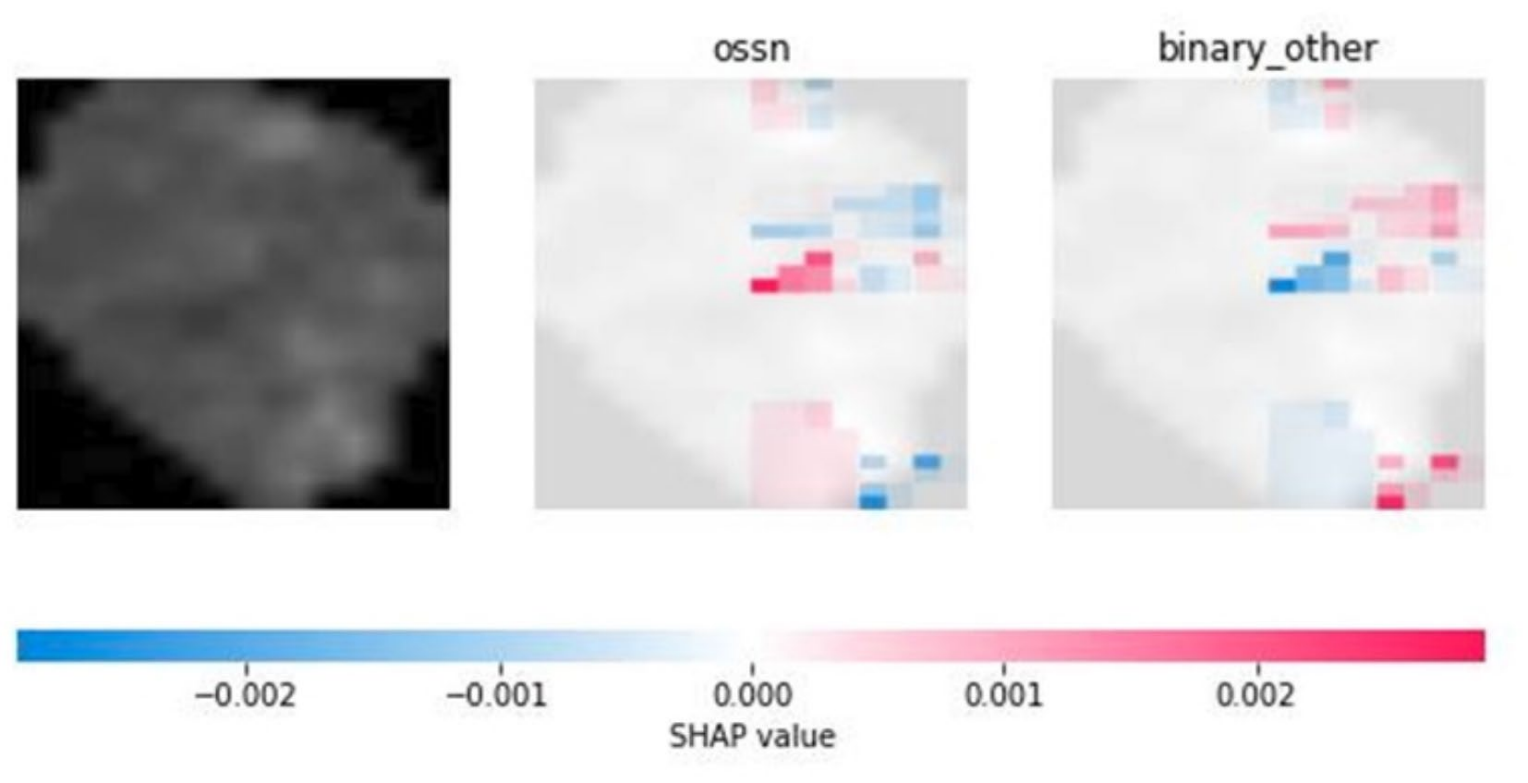
Example of visualizing Shapley evaluation of an OSSN cell.

**Fig. 7:**
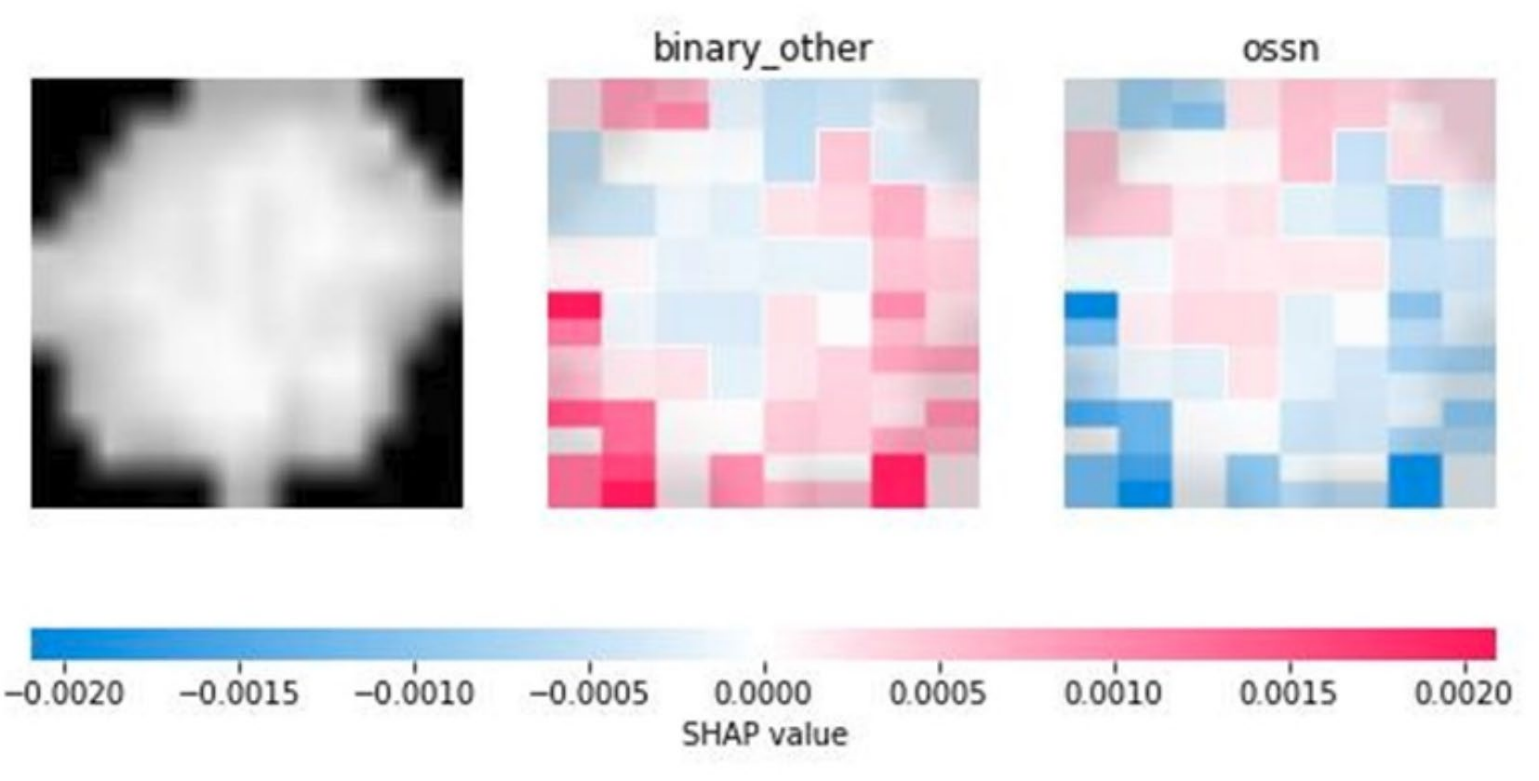
Example of visualizing Shapley evaluation of a non-OSSN cell.

After analyzing the Shapley values of the cell images, we found that the Shapley values are relatively low, they are below 0.1. It implies that the sensitivity of the model must be further evaluated. Also, it indicates that no single pixel dramatically influences the model’s predictions. The decision-making process of the model is based on a collective assessment of the pixels rather than being heavily reliant on specific regions of the image. However, based on the evaluations, the most important parts seem to be the edges of the cells and the region of the nucleus. Both the edge of the cells and the region of the nucleus may have significant color and shape information on a pixel level, so it indicates that a single pixel cannot have high power of decision (Figs. 6 and 7).

#### Dimension reduction

**Fig. 8:**
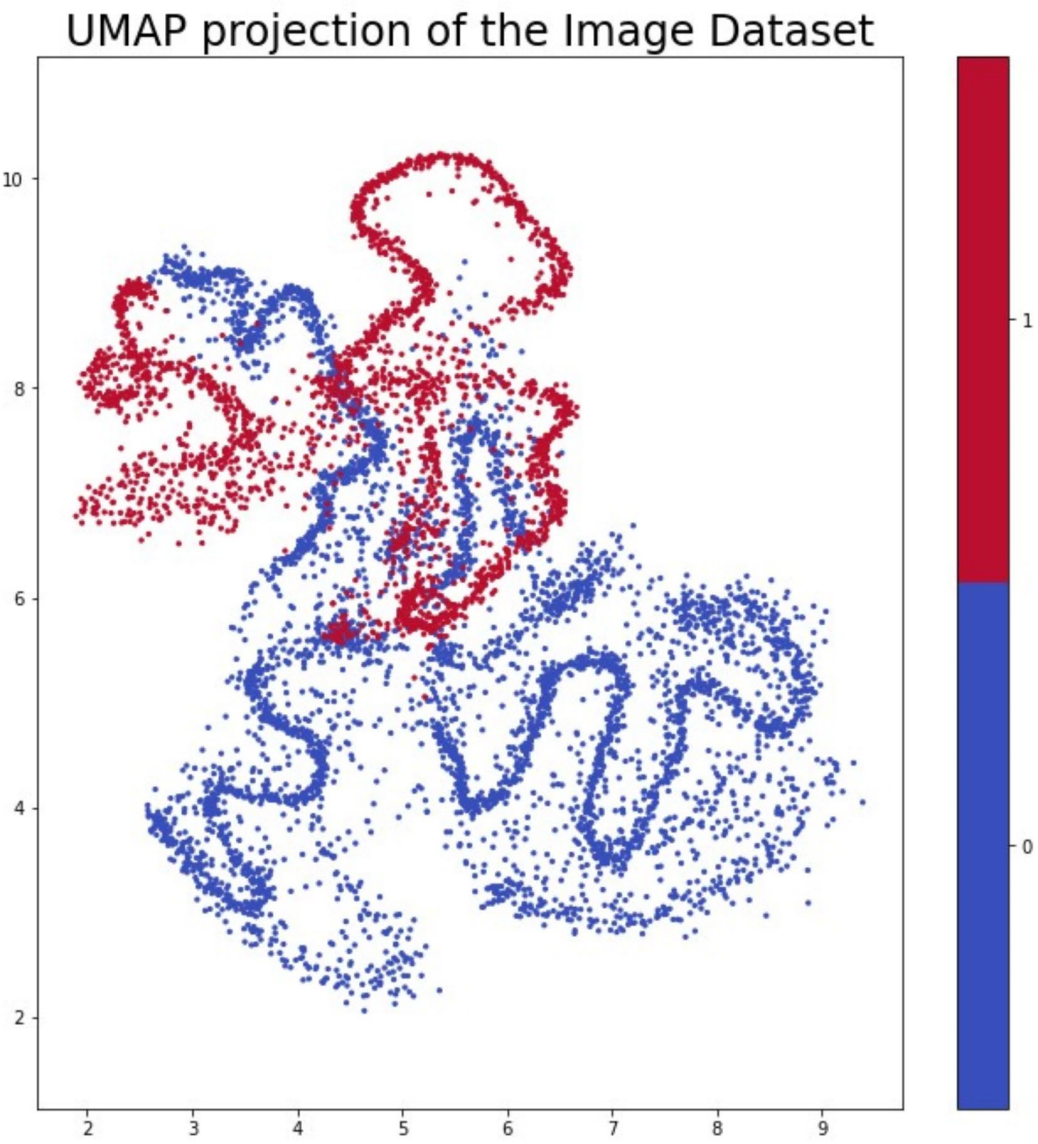
The result of UMAP projection using Yule distance function.

The most important results were obtained using the complex distance function metrics. Our most successful cell-classifier model makes a binary evaluation, so it is not surprising that the metrics for binary data are working the best, however the simplest distance functions like the Hamming and Jaccard distance functions did not show significant dissimilarity between the vectors. In this analysis, we made an evaluation with the Yule distance function. Figure 8. depicts the result of UMAP projection using Yule distance function. We can see distinct clusters but there is an overlap that suggests that our neural network’s Shapley values provide clear and consistent insights into its decision-making process. However the success of the complex distance function indicates that there is no dedicated feature that influences the decision, rather there is a composite feature set that helps in accurate classification.

**Fig. 9:**
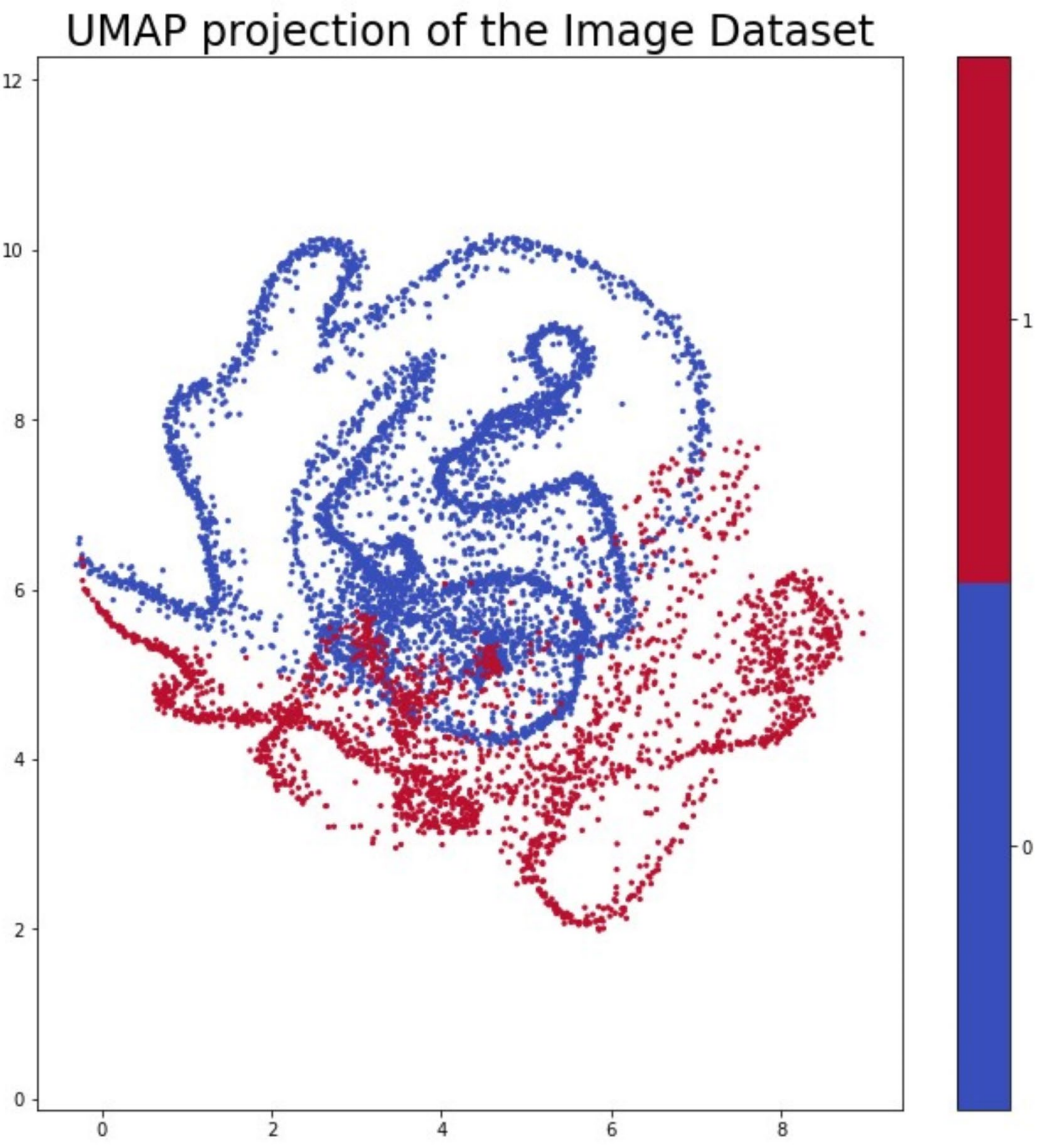
The result of UMAP projection using Russell-Rao distance function.

Another successful UMAP projection analysis was made using the Russell-Rao distance function Unlike the Yule distance, the Russell-Rao distance is based on the count of matches between the two vectors but focuses primarily on the “True” matches. In Fig. 9, we can see that we have well-defined clusters with an overlap. This overlap confirms that the features do not always provide a strong separation.

#### Grad-CAM and feature importance analyses

**Fig. 10:**
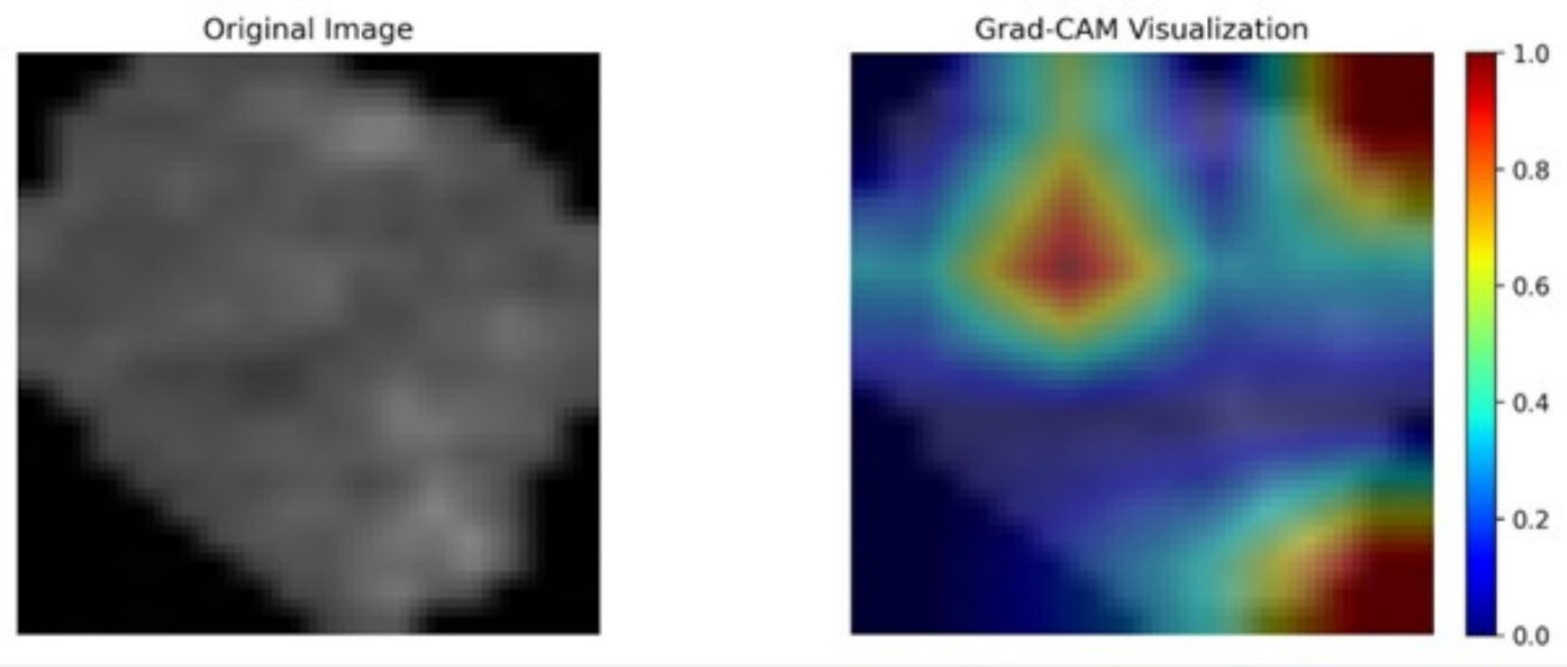
Grad-CAM analysis of an OSSN cell.

**Fig. 11:**
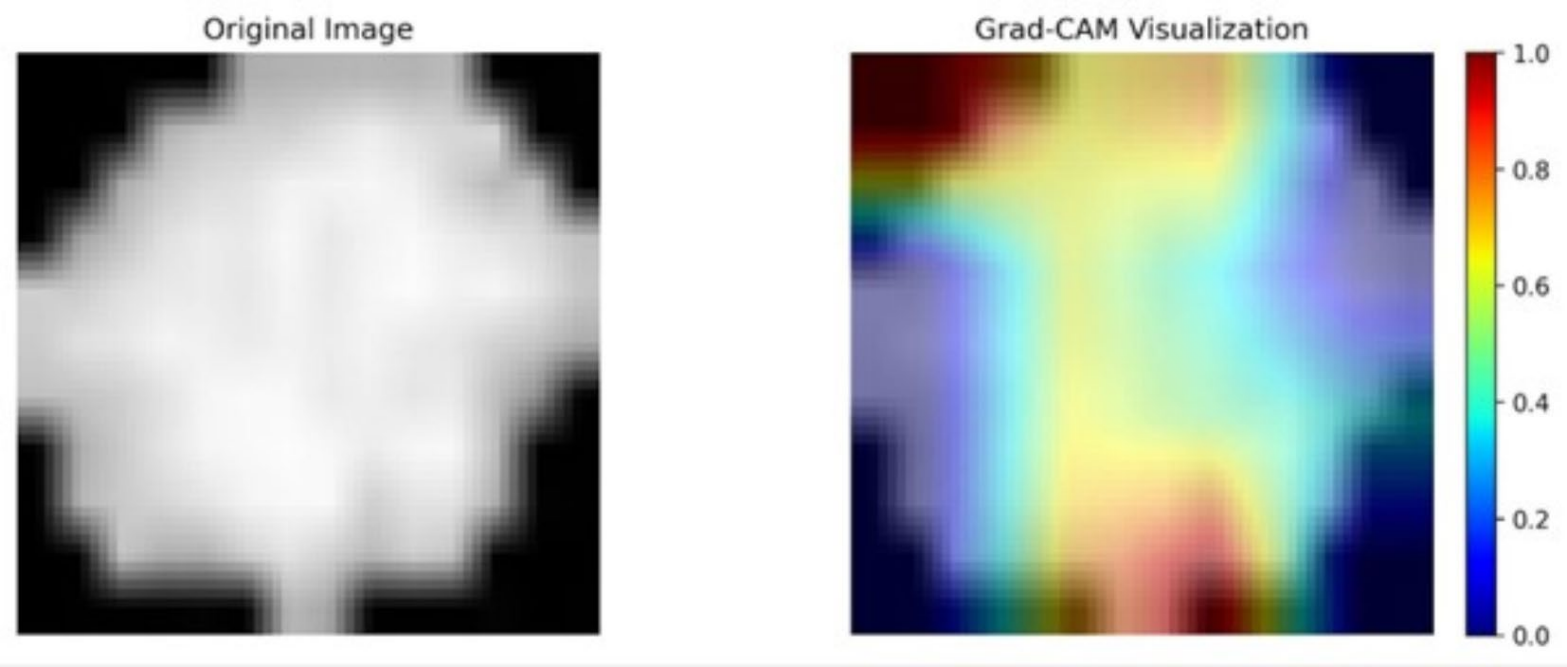
Grad-CAM analysis of a non-OSSN cell.

The results of Grad-CAM analysis applied to an OSSN and a non-OSSN cell are illustrated in Figs. 10 and 11. The neural network’s activation regions are represented by the crimson zones. The results of the Shapley analysis and the Grad-CAM analysis show similarities. It is evident that the margins and centroids of the cells are crucial in the decision-making process.

**Fig. 12:**
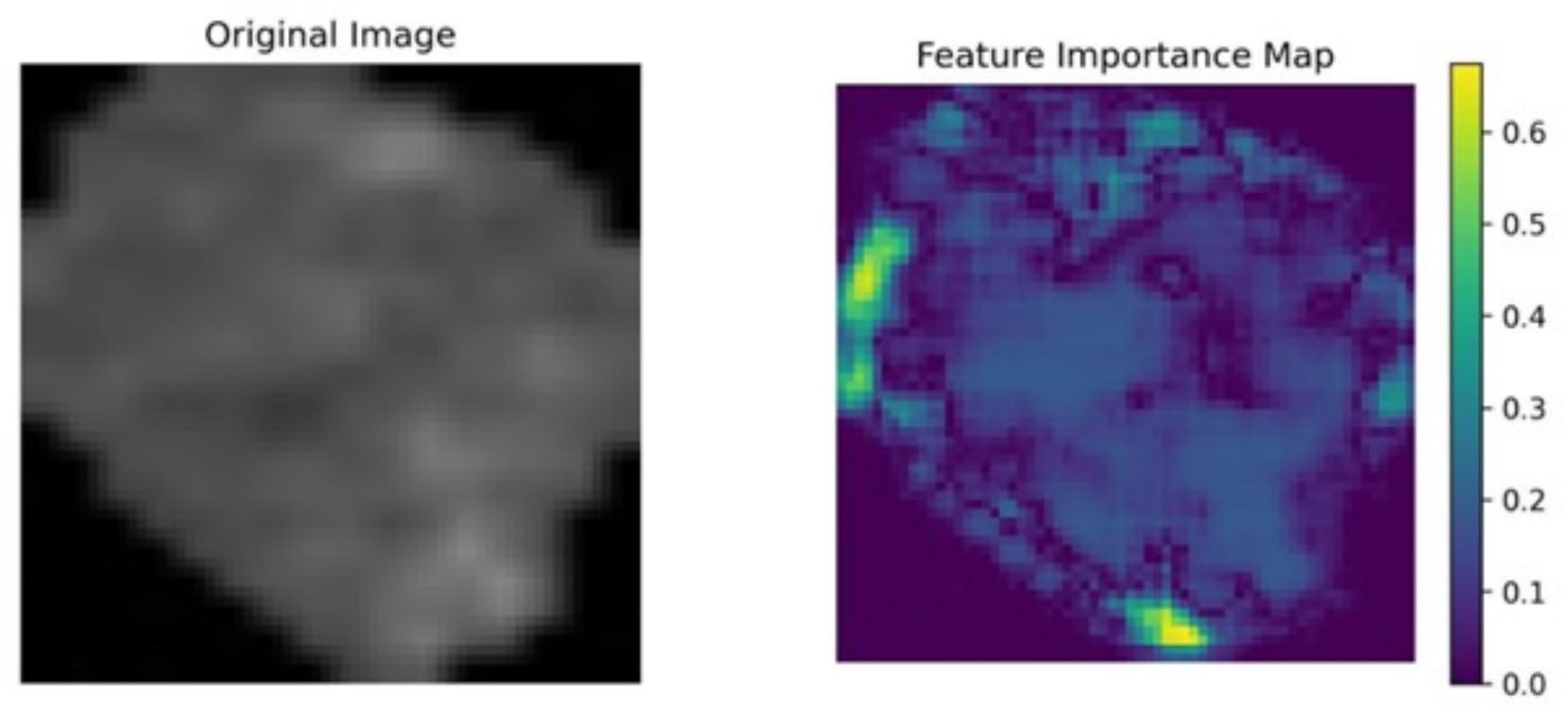
Feature importance analysis of an OSSN cell.

**Fig. 13:**
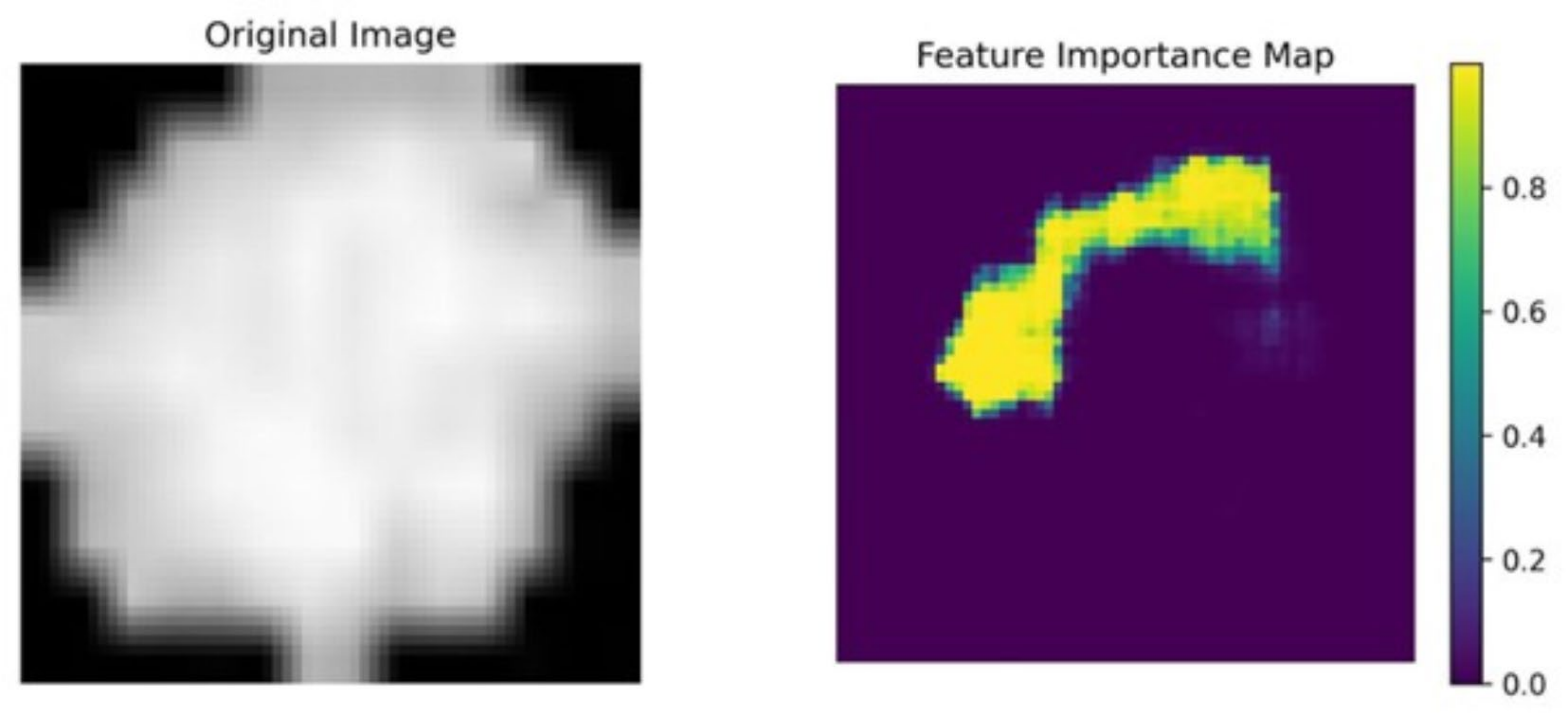
Feature importance analysis of an OSSN cell.

The findings of the feature importance analysis are illustrated in Figs. 11, 12 and 13. On the right, the feature importance maps indicate which pixels are crucial in the decision-making process. In this case, we examine the pixels individually; however, it is evident that the margins and central regions are essential for decision-making.

Naturally, the differences between the various analysis techniques are apparent as the analytical approaches are completely differing and they assess different relationships and significances. The implications that have been stated through the assessment of Shapley values are substantiated by these additional methods.

## Discussion

Although OSSN is a rare disease, its incidence is on the rise in several countries affecting an increasingly younger population. Diagnosing OSSN based on clinical presentation can be challenging due to the varying appearance of the lesions^30^. Although the gold standard diagnostic technique is still excisional biopsy using the “no-touch technique”, non-invasive imaging modalities including AS-OCT, ultrasound biomicroscopy (UBM) and IVCM play an important role in the diagnosis and treatment^31^. Ocular surface diseases with similar appearances can be differentiated using IVCM which allows for visualization of cellular structures of the cornea and conjunctiva and the subbasal nerve fibers in 800x magnification^32^. During the examination, hundreds of IVCM images can be taken of the ocular surface abnormalities and healthy corneal layers. The process of evaluating and interpreting these images is time-consuming and requires expertise.

As IVCM is a non-invasive diagnostic modality that allows for recording high-quality images in large quantities, it serves as excellent training data for AI models^33^. Several studies focus on the corneal nerve fibers and developed different segmentation models, such as the widely used ACCMetrics software (University of Manchester, Manchester, UK)^34^. Our AI model was trained to detect the specific IVCM characteristics of OSSN: „starry sky” pattern, enlarged, irregular epithelial cells, hyperkeratosis and mitosis^35^. The model can process large amounts of data in a shorter time and propose a diagnosis with high accuracy.

In this paper, we showed an AI-based low-level technique to differentiate OSSN lesions from other diseases identified on the ocular surface with high precision; however, the tested AI models were not efficient in the OSSN clinical signs classification problem. This is due to the unbalanced dataset and the few number of samples in some classes. Mitosis is a very rare symptom and we had only 3 samples from that class. Furthermore, hyperkeratosis was only present on 27 images. To improve the accuracy of our models, we divided the rectangle shaped signs into squares, thus we had more samples that still contained the required image information to identify the alteration. This technique significantly improved the accuracy of the models. During the analysis of sign-based classification results, we found that the models are mixing the starry sky and the irregularly shaped cells classes. Starry sky is considered as a group of irregularly shaped cells with increased size of nucleus, so irregularly shaped cells form a subclass of starry sky. For starry sky and irregularly shaped cells classes, we have to cleanse the dataset and assign the appropriate label to the samples to clearly distinguish the two classes. We also found that the trained models were not able to correctly classify the mitotic cells. To improve the accuracy in the class of mitosis, we introduced the technique called few-shot learning^36^. With few-shot learning, we correctly classified our validation data of mitosis class. Our experience is that OSSN sign classification is a task that is difficult to manage with a generic model. The results can be better if we use different techniques for the different signs and not make confusing classifying even if the characteristics of the classes are very similar to each other.

One of the most conspicuous findings of our study was that IVCM images of healthy subjects and patients with different ocular surface disease may hold patient-specific features. Since IVCM generates high-resolution images of living tissues at a cellular level, it offers a unique window into patient-specific biological features. The high degree of spatial detail captured by IVCM makes the images particularly distinctive, reflecting individual variability in tissue architecture, cellular arrangement, and molecular expression. This patient-specificity arises from the inherent biological differences in cell morphology, density, and tissue structure in health and disease. As a result, IVCM images can act like a “fingerprint” for each individual, potentially enabling personalized diagnostics, monitoring, and treatment planning. This approach holds promise for precision medicine, where unique tissue characteristics could be used to guide more tailored interventions and track disease progression on an individual basis.

By leveraging Shapley values, researchers and practitioners are able to demystify the black-box nature of sophisticated neural networks, facilitating a clearer insight into their operations and enhancing trust in AI systems. This approach not only enhances transparency but also aids in identifying biases, improving model robustness, and guiding the development of more interpretable AI models^37^. Upon analyzing the Shapley values related to our model we found that the model’s decision-making relies on an overall assessment of all pixels in the image, with the edges of the cells and the nucleus being the most important areas; thus, no single pixel has a strong influence on the outcome.

As the Shapley values showed that it is not trivial which parts of the cells play the most important roles in decision making. Analyzing the UMAP projections we found that the efficacy of the complex distance function suggests that there is no singular feature that significantly drives the decision-making process. Instead, the results indicate the presence of a composite feature set that collectively contributes to accurate classification. In summary, UMAP serves as a powerful tool in the arsenal of explainable AI, bridging the gap between the opaque, complex computations of neural networks and the need for understandable, interpretable AI solutions^38,39^.

Although AI can be very useful in making objective clinical decisions it still has some limitations. For accurate training, AI models need a large amount of high-quality training data. Without sufficient data, their performance can be limited^40^. The images captured by different types of devices may differ in quality, resolution and color. These discrepancies can influence diagnostic accuracy. AI algorithms can acquire biases from the data used for training, resulting in biased decisions and incorrect diagnostic outcomes. Understanding the reasons behind predictions or decisions of some AI models can be challenging due to their lack of interpretability^41^.

Our study had some limitations, including the small training dataset and the lack of diversity in the non-OSSN lesions. Ocular surface diseases can manifest in various ways, making them challenging to diagnose accurately based on IVCM images alone. Moreover, even in patients with OSSN, the images of the normal corneal and conjunctival regions may appear completely normal, which can complicate the differentiation between pathological and healthy tissues. This highlights the critical challenge of image acquisition and the need for precise targeting of affected areas during the examination process^35^. Future studies need a multi-center design to collect a large clinical dataset and include various ocular surface abnormalities. Furthermore, conducting thorough validation on external datasets and prospective clinical studies is essential for evaluating the model’s performance and ensuring its successful implementation in clinical practice.

## Conclusion

We established a technique and a deep learning model for detecting alterations on IVCM images characteristic of OSSN having a small dataset. Our network demonstrated high accuracy in binary classification, pattern recognition and cellular-level classification. We showed various techniques to improve the size of the training dataset, both for pattern recognition and cell-level classification tasks. Due to the accurate cell segmentation approach, we identified the thousands of cells in our dataset and trained a VGG19 classification model. We also made comparisons of different neural networks to find the best working one. We successfully classified the healthy and unhealthy cells on all IVCM images and found that the cell-level classification results can be back propagated to image-level and patient-level as well. The different aggregation steps further improved the classification and we achieved 100% accuracy in patient-level classification. We validated and explained our results using Shapley values, and we showed that the classification problem is more complex than a simple shape or color differentiation using UMAP projection. Our observations and implications were verified through a Grad-CAM and a feature importance analysis. The CORN datasets were used to assess the clinical applicability of our methodologies. As far as we know, this study represents the first attempt to develop and evaluate artificial intelligence models for detecting OSSN using IVCM images at different levels with a small dataset. We found that even the cell-groups can be patient-specific and it is important to construct the training and validation sets with appropriate split of dataset.

AI-enhanced analysis of IVCM images shows strong potential in improving the diagnosis of OSSN, particularly in distinguishing between healthy and abnormal tissues. However, feature classification remains challenging, and future studies require larger datasets and refined techniques. The patient-specific information within IVCM images offers promise for personalized diagnostics and treatment monitoring in ocular oncology.

## Data Availability

The data that support the findings of this study are available from the corresponding author upon reasonable request.
